# Dietary Supplementation with Sugar Beet Fructooligosaccharides and Garlic Residues Promotes Growth of Beneficial Bacteria and Increases Weight Gain in Neonatal Lambs

**DOI:** 10.3390/biom10081179

**Published:** 2020-08-13

**Authors:** Narciso M. Quijada, Raúl Bodas, Jose M. Lorenzo, Stephan Schmitz-Esser, David Rodríguez-Lázaro, Marta Hernández

**Affiliations:** 1 Laboratorio de Biología Molecular y Microbiología, Instituto Tecnológico Agrario de Castilla y León (ITACyL), 47071 Valladolid, Spain; nmartinquijada@gmail.com (N.M.Q.); bodrodra@itacyl.es (R.B.); 2 Division of Microbial Ecology, Centre for Microbiology and Environmental Systems Science, University of Vienna, 1090 Vienna, Austria; 3 Institute of Food Safety, Food Technology and Veterinary Public Health, Unit of Food Microbiology, University of Veterinary Medicine Vienna, 1210 Vienna, Austria; 4 Centro Tecnológico de la Carne de Galicia, Avd. Galicia nº 4, Parque Tecnológico de Galicia, San Cibrao das Viñas, 32900 Ourense, Spain; jmlorenzo@ceteca.net; 5 Área de Tecnología de los Alimentos, Facultad de Ciencias de Ourense, Universidad de Vigo, 32004 Ourense, Spain; 6 Department of Animal Science, Iowa State University, Ames, IA 50011-3150, USA; sse@iastate.edu; 7 Microbiology Division, Department of Biotechnology and Food Science, Faculty of Sciences, University of Burgos, 09001 Burgos, Spain; drlazaro@ubu.es

**Keywords:** gut microbiota, prebiotics, high-throughput sequencing

## Abstract

The proper development of the early gastrointestinal tract (GIT) microbiota is critical for newborn ruminants. This microbiota is susceptible to modification by diverse external factors (such as diet) that can lead to long-lasting results when occurring in young ruminants. Dietary supplementation with prebiotics, ingredients nondigestible and nonabsorbable by the host that stimulate the growth of beneficial GIT bacteria, has been applied worldwide as a potential approach in order to improve ruminant health and production yields. However, how prebiotics affect the GIT microbiota during ruminants’ early life is still poorly understood. We investigated the effect of milk supplementation with a combination of two well-known prebiotics, fructooligosaccharides (FOS) from sugar beet and garlic residues (all together named as “additive”), exerted on preweaned lamb growth and the composition of their fecal microbiota, by using 16S rRNA gene amplicon high-throughput sequencing. The results showed a significant increase in the mean daily weight gain of lambs fed with the additive. Lamb fecal microbiota was also influenced by the additive intake, as additive-diet lambs showed lower bacterial diversity and were significantly more abundant in *Bifidobacterium*, *Enterococcus*, *Lactobacillus* and *Veillonella*. These bacteria have been previously reported to confer beneficial properties to the ruminant, including promotion of growth and health status, and our results showed that they were strongly linked to the additive intake and the increased weight gain of lambs. This study points out the combination of FOS from sugar beet and garlic residues as a potential prebiotic to be used in young ruminants’ nutrition in order to improve production yields.

## 1. Introduction

Sheep and lamb farming is a practice widespread worldwide due to the appreciated value of the associated products (meat, milk and wool) and great efforts are continuously made to select the best breeds and to improve production yields and performance [1]. Sheep are grazers and their diet consist largely of plant cell-wall polysaccharides as an energy source [2]. However, most dietary polysaccharides cannot be degraded by the animal, and, instead, a wide variety of microorganisms that inhabit the gastrointestinal tract (GIT) are the responsible of the degradation of these polymers into volatile fatty acids that can be therefore assimilated by the host [3,4]. The composition of the GIT microbiota differs widely depending on the location within the ruminant GIT and needs to be investigated independently [5,6]. Whereas the rumen microbiota, which is the main one responsible for the plant cell-wall degradation, has been well characterized, other segments of the ruminant GIT are still poorly known [5,6,7,8].

Neonatal and preweaned lambs possess an immature rumen, and so, milk bypasses rumen to the abomasum and reaches the intestine where, as in nonruminant mammals, its microbiota exerts a key role in metabolism, physiology and immunity [9,10,11,12,13]. The microbial colonization of the GIT of neonatal ruminants starts at birth and the microbial communities evolve widely during the first weeks of life [14,15,16,17,18,19]. The early life of ruminants is pivotal for the establishment of a functional GIT microbiota, as it is influenced by multiple factors including age, diet, feeding method, gender and weather [20,21]. Diet has been shown to have a significant impact on GIT microbiota structure and has the potential to be used in order to manipulate it towards specific outcomes [22]. Unlike mature ruminants, whose GIT microbiota is relatively stable to changes, several studies highlighted the effect of diet in shaping the GIT microbiome of lambs during their early life [4,22,23,24,25,26]. There is evidence that a window exists during the first weeks of life in which the GIT microbiota is more susceptible to changes and where manipulation through diet could lead to long-lasting results [22,27,28].

Feeding of lambs with sheep breast milk provides them with beneficial compounds for their survival and optimal development, including oligosaccharides and glycans that stimulate the growth of beneficial GIT microbiota, such as *Bifidobacterium* and lactic acid bacteria (LAB) [10,29]. The feeding method is a critical factor for lamb GIT microbiota development and the beneficial effects of breast-feeding over bottle-feeding have been stated [21]. However, in many commercial dairy sheep farms, where sheep milk is used for other purposes, lambs are often separated from their mothers after birth and are fed with milk replacer [23].

All the facts stated above indicate that the manipulation of young ruminant GIT microbiota based on dietary conditions represents a potential tool towards improving ruminant health and production yields. Several attempts, such as the dietary supplementation with prebiotics, have been applied worldwide towards this aim [30,31]. Prebiotics are ingredients nondigestible and nonabsorbable by the host but that can be fermented by certain GIT microbes, therefore stimulating their growth [32]. That is the case of fructooligosaccharides (FOS), which are soluble fibers naturally present in some vegetables, such as sugar beet, whose inclusion in the diet have been related to a positive effect on feed efficiency and a reduced incidence and severity of enteric diseases [30,33,34,35,36]. Besides, garlic (*Allium sativum*) is a plant that has been used for a long time for both culinary and medical purposes by many cultures, due to its antimicrobial, antioxidant and immunostimulating properties [37,38]. The complex composition of garlic involves a paradoxical result on the GIT microbiome [39]. Garlic is famous for its antimicrobial properties, mainly due to its composition of organosulfur compounds, such as allicin, that have shown a bactericidal effect against *Campylobacter jejuni*, *Enterococcus faecalis*, *Escherichia coli*, *Neisseria gonorrhoeae*, *Salmonella typhimurium* and *Staphylococcus aureus* [38,40,41,42]. On the other hand, garlic is rich in nondigestible polysaccharides, such as fructans, that act as a prebiotic for specific GIT microbiota [43].

In this study, we aimed to investigate the effects of preweaned lamb dietary supplementation with a combination of sugar beet FOS and garlic residues (derived from the food industry and all together named as “additive”) on lamb growth and the composition of their GIT microbiota. Reutilization of alimentary industry residues for animal nutrition is valuable to contribute to circular economy as far as their biological effect can be also demonstrated more than the economic gain. The lambs selected in this study were grown in a farm where the sheep breast milk is derived from milk for cheese production and, therefore, the lambs are usually fed with milk replacer. Consequently, the lamb meat of the animals included in the study are intended for human consumption. This study is an experimental work carried out in field conditions to evaluate the changes in the gut microbiota of lambs submitted to a different diet including prebiotics such as fructooligosaccharides (FOS) and garlic. Thus, in order to analyze GIT microbiota of lambs in a noninvasive manner, DNA was extracted from fecal samples and subjected to 16S rRNA gene amplicon high-throughput sequencing (HTS) to observe significant effects of the diet.

## 2. Materials and Methods

### 2.1. Ethics Statement

All handling practices followed the recommendations of the Directive 2010/63/EU of the European Parliament and of the Council on the protection of animals used for scientific purposes and the ITACyL Animal Experimentation Ethics Committee.

### 2.2. Sampling and Experimental Setup

All lambs (*Ovis aries*, “Assaf” breed) were born and grown in the AGM farm dedicated to sheep production (Olmedo, Spain) and devised for human consumption and resources optimization. Sheep breast milk is derived from the milk industry and for cheese production. Therefore, neonatal lambs are usually fed with milk replacer (24.0% crude protein, 24.6% ether extract, 6.8% ash, air-dry powder basis; Novilam 63 COC; Schils bv, Sittard, the Netherlands) at 16% (*w*/*w*) in warm water (37 °C).

In this study, the newborn lambs were separated from their mothers at birth and divided into two different groups in two different compartments within the farm. No contact between lambs of both groups or with their mothers occurred throughout the study. Each group of lambs followed a different diet. The first group of lambs, named as “control-diet group”, was fed with the milk replacer described above. The second group of lambs, named as “additive-diet group”, was fed with the milk replacer mix with a combination of commercially available fructo-oligosaccharides (FOS) from beet (BETAFOS^60^), at a final concentration of 11.4%, and garlic residues (altogether called as “additive” in this study). The formulation and specific concentration of the ingredients of this additive belongs to the line Prebionat from AB Azucarera Iberia (Madrid, Spain), that is currently patent pending. Three ml of this additive solution was added per 1 L of milk replacer. Milk was taken by lambs ad libitum from a milk tank. As a mean, 43 L of milk was taken per lamb throughout the study, without significant differences between control- and additive-diet groups. At early times of the ongoing study, several lambs died or required an antibiotic treatment, consequently being excluded from the study. At the end of the study, control- and additive-diet groups were composed by 19 and 15 lambs, respectively. Lambs were weighed throughout the study to calculate the mean daily weight gain. In order to investigate lamb GIT microbiota, deep rectal swabs from lambs were collected after 25 days of feeding either control or additive diets. For the quantification of serum immunoglobulins, blood samples were taken at two time points (days 2 and 16) by venepuncture in the jugular vein using EDTA-containing vacutainer tubes. Blood samples were then centrifuged at 1000× *g* for 15 min at 6 °C within 30 min after extraction. Plasma was collected and stored at −20 °C until analysis. Sheep immunoglobulin A, G and M were analyzed by using commercial ELISA kits following manufacturer’s instructions (Cusabio Technology LCC, Houston, TX, USA). All samples were taken in the most careful conditions as possible in order to not disturb the animals whose final destination was human consumption.

### 2.3. Total DNA Extraction, 16S rRNA Gene Amplicon Library Preparation and Sequencing

Total DNA was extracted from each stool sample using the QIAamp DNA Stool Mini Kit (Qiagen, Hilden, Germany), according to manufacturer’s instructions. The DNA concentration was determined using a Qubit^®^ fluorimeter (Invitrogen, Carlsbad, CA, USA). Microbial diversity was studied by sequencing the amplified V3-V4 region of the 16S rRNA gene by using primers and PCR conditions previously reported [44]. Sample multiplexing, library purification, and sequencing were carried out as described in the “16S Metagenomic Sequencing Library Preparation” guide by Illumina.

Libraries were sequenced on a MiSeq Illumina platform at the University of Burgos (UBU, Burgos, Spain), leading to 300 bp, paired-end reads.

### 2.4. Bioinformatics and Data Analysis

Raw demultiplexed sequence data were processed using the QIIME2 version 2018.4 pipeline [45]. q2-dada2 [46] and q2-feature-table [47] plugins were used for quality filtering of the reads, merging of the paired ends, chimera removal and identification of Amplicon Sequence Variants (ASVs). Quality of the 16S rRNA gene amplicon dataset was evaluated prior and after the trimming step by using FASTQC (version 0.11.8, http://www.bioinformatics.babraham.ac.uk/projects/fastqc/). ASVs rely on single nucleotide differences between sequences and can be considered as Operational Taxonomic Units (OTUs) clustered at 100% identity threshold. Good quality 16S rRNA gene amplicon sequences ranged from 23,761 to 192,558 reads per sample. A phylogenetic tree was built using q2-alignment [48] and q2-phylogeny [49] plugins. A pretrained Naïve Bayes classifier based on the SILVA database [50], previously trimmed to harbor the V3-V4 region of 16S rRNA gene, was used for taxonomy assignment of the identified ASVs by using the q2-feature-classifier plugin [51]. Alpha- and beta-diversity were analyzed by using q2-diversity (https://docs.qiime2.org/2018.4/plugins/available/diversity/) and q2-taxa (https://docs.qiime2.org/2018.4/plugins/available/taxa/) plugins. For beta-diversity studies, samples were rarefied to 23,761 reads per sample, in order to avoid biases due to different sequencing depths, and weighted UniFrac distances [52] were calculated. Plotting was carried out in R environment (v3.6.1, https://www.r-project.org) using *dplyr* (v1.0.0), *ggplot2* (v3.3.0), *made4* (v1.60.0) and *rehsape2* (v1.4.3) packages.

Normal distribution of data was evaluated by the Shapiro–Wilk’s test, and statistical Wilcoxon test analysis or unpaired two-samples *t*-test were performed for normally and non-normally distributed data, respectively, by using R packages *dplyr* (v1.0.0)*, ggpubr* (v0.2.5) and *multcomp* (v1.4.13). Results were considered significant when *p* value < 0.05. Principal Component Analysis (PCA) of the most abundant ASVs in lamb feces was performed in R environment using *FactoMineR* (v2.3) and *factoextra* (v1.0.7) packages. Permutational multivariate analysis of variance (PERMANOVA) was performed to investigate the significance of the differences observed in control- and additive-diet groups at genera level by using the *vegan* (v2.5.6) package. Linear discriminant analysis effect size (LEfSe) [53] was performed to determine differentially abundant taxa and ASVs between control- and additive-diet groups of animals.

## 3. Results

### 3.1. Effect of the Diet on Weight Gain and Blood Immunoglobulin Concentration of Lambs

**Figure 1:**
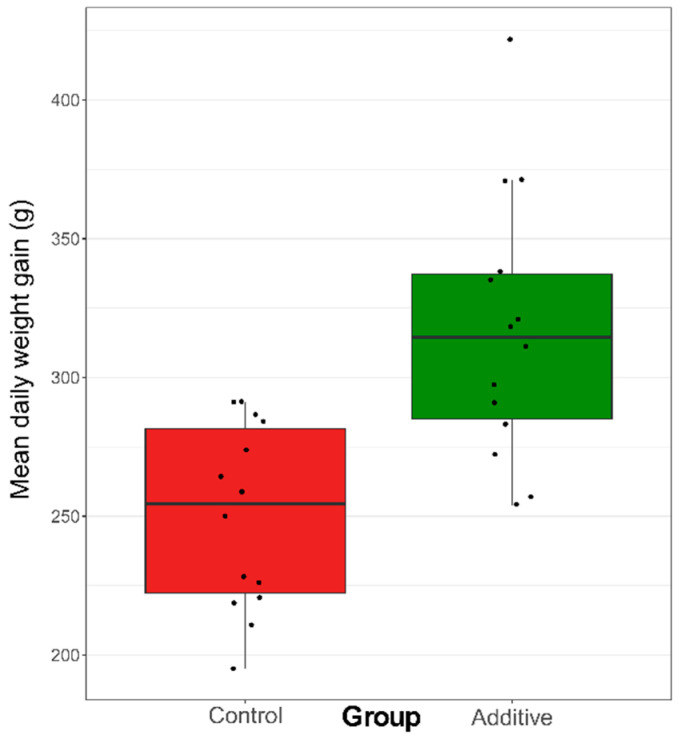
Box plot showing the mean daily weight gain (g) of the different lambs (black dots) that followed control (red) or additive (green) diets. The height of each box ranges from the first (Q1) to the third (Q3) quartile, which are the median values of the lower or the upper half of the mean daily weight gain (g), respectively. The horizontal black line inside each box corresponds to the median value (Q2) of the mean daily weight gain (g) of each group. Differences between the mean daily weight gain (g) between control- and additive diet lambs were statistically significant (*p* < 0.05).

The lambs that were fed with the additive showed a significant increase in the mean daily weight gain at the end of the study in comparison to the control-diet lambs (*p* < 0.05, Figure 1 and Supplementary File 1). This increase in the mean daily weight gain of lambs was not related to an increased appetite, as both groups of lambs received similar amounts of milk (data not shown). Additionally, no differences were observed in blood immunoglobulin concentrations regarding the additive intake (Supplementary File 1).

### 3.2. Sequencing Depth, Alpha Diversity and Lamb Fecal Bacteria Composition

Sequence analysis of the 34 lamb fecal samples resulted in 3,880,330 sequences after quality control, chimera removal and paired-end joining, with a median of 112,721 (±49,401) sequences per sample. With a subsample of 23,761 sequences per sample, all the sample-based rarefaction curves reached a plateau (data not shown) and Good’s coverage index was almost one for all samples (Supplementary File 2), suggesting that the bacterial diversity was sufficiently covered by our sequencing depth. Overall, 1844 unique Amplicon Single Variants (ASVs), based on single nucleotide differences between sequences, were identified. Of these, 394 ASVs showed an overall abundance of over 0.01% and 22 ASVs showed an overall relative abundance greater than 1%. The ASVs were classified into 15 phyla, where *Firmicutes* (54.4% and 55.7% relative abundance in control- and additive-diet lamb fecal samples, respectively), *Bacteroidetes* (18.9% and 16.7%), *Actinobacteria* (14.9% and 16.3%) and *Proteobacteria* (10.9% and 7.1%) were the most abundant overall.

**Figure 2:**
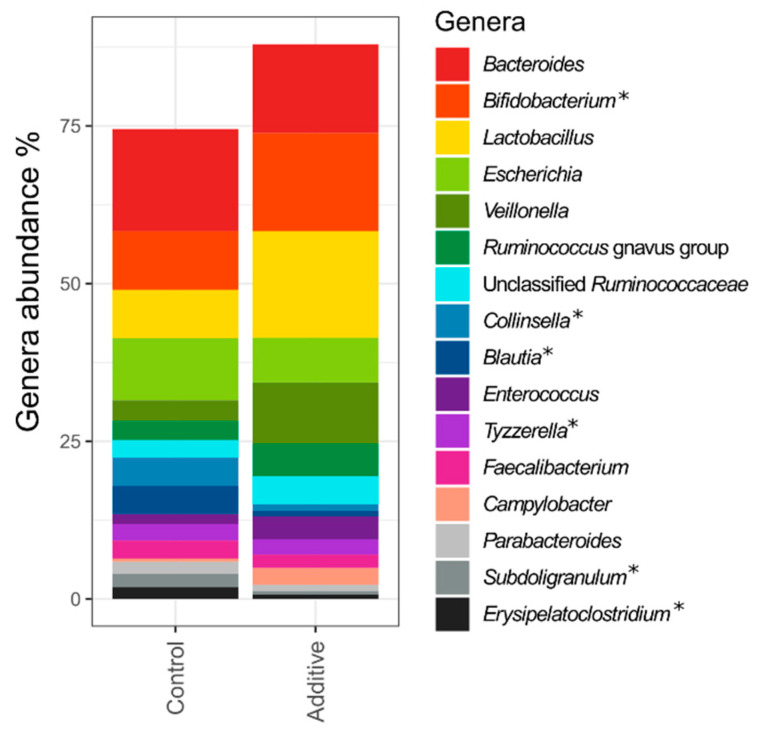
Relative abundance of the 16 most abundant genera found overall in control- or additive-diet lamb fecal samples. Asterisks point out those genera that showed significant differential abundance between control- and additive-diet lamb fecal samples, according to LEfSe analysis (*p* < 0.05).

The three most abundant ASVs overall were assigned to *Bifidobacterium*, *Escherichia* and *Veillonella*, and were present in all lamb fecal samples and accounted for 10.6%, 5.9% and 5.7% of all the 16S rRNA gene sequences, respectively. Figure 2 shows the relative abundance of the 16 most abundant genera overall in control- and additive-diet lambs. Differences can be observed between the two groups—additive-diet lambs fecal microbiota showed a higher relative abundance of *Bifidobacterium*, *Lactobacillus* and *Veillonella*, whereas control-diet lambs fecal microbiota harbored greater relative abundance of *Escherichia*, *Collinsella* and *Blautia*. PERMANOVA analysis at genera level showed statistically significant differences between the bacterial genera identified in control- and additive-diet lambs’ feces (*p* value = 0.003). Alpha-diversity metrics (Chao1, Shannon and Simpson) were analyzed revealing higher richness and diversity in control-diet lamb fecal samples (Supplementary File 2).

**Figure 3:**
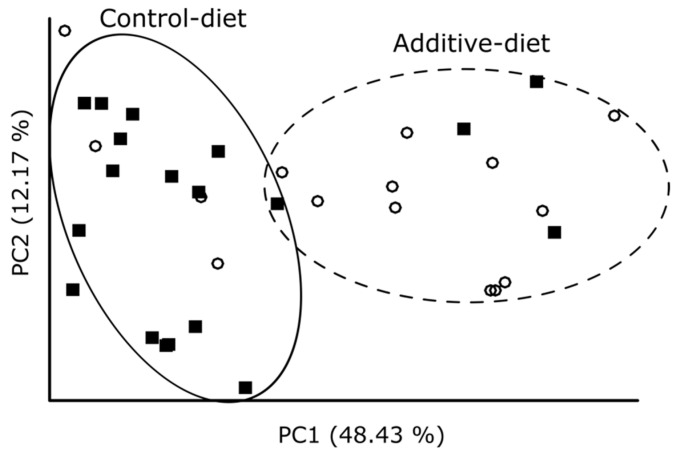
Principal Coordinates Analysis (PCoA) of the 16S rRNA gene amplicon Amplicon Single Variants (ASVs) based on Weighted UniFrac distances. White circles represent additive-diet lamb fecal samples whereas black squares represent additive-diet ones.

β-diversity (differences between samples based on their microbial community composition) was evaluated by Weighted UniFrac and represented as a Principal Coordinates Analysis (PCoA) in Figure 3. Differences between both groups of lamb fecal samples can be observed, as they tended to cluster together according to the diet followed by each group of lambs.

### 3.3. Evaluation of the Effect of Additive Intake over Lamb Fecal Microbiota

**Figure 4:**
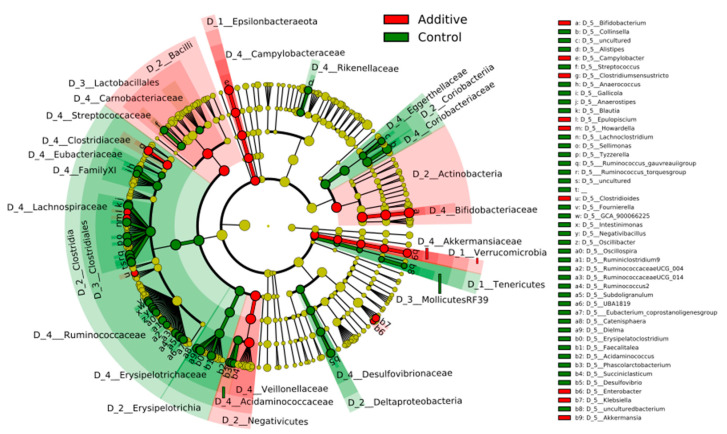
Cladogram reflecting statistically significant differences (calculated by LEfSE) of different taxa found in lamb feces between additive (red) and control (green) diets. Circles represent the different taxa in the different levels of taxonomy. From the inner to the outer circle—phylum (D_1), class (D_2), order (D_3), family (D_4), genus (D_5).

In order to evaluate the differences in the abundance of fecal bacterial taxa between additive- and control-diet lambs, LEfSe statistical analysis was performed and represented as a cladogram in Figure 4. The cladogram shows the different bacterial taxa that were identified in the study, colored in red or green if they were significantly more abundant in additive-diet or control-diet lamb fecal samples, respectively. It can be observed that *Bifidobacterium* (genus), *Veillonellaceae* (family) and *Lactobacillales* (order) are statistically significantly more abundant in additive-diet lambs whereas *Clostridiales* (order), *Ruminococcaceae* (family) and *Erysipelotrichaceae* (family) were more abundant in control-diet lambs.

**Figure 5:**
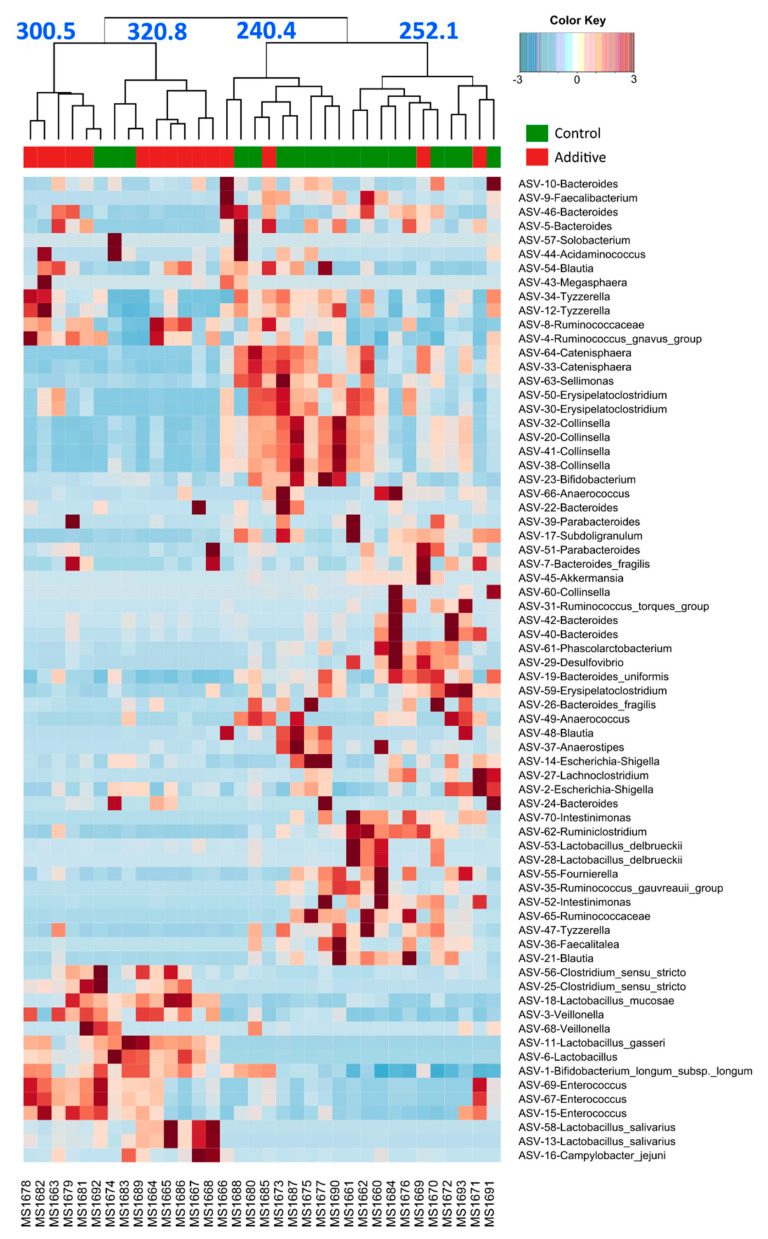
Heatmap showing the distribution of the 70 most abundant ASVs (ASV appear in the y-axis at the right side of the figure with their taxonomic assignation according to the SILVA database) in control- (green) or additive- (red) diet lamb fecal samples. The heatmap ranges from dark blue (less occurrence) to dark red (more occurrence), indicating if the different ASVs were more occurrent and abundant in the different samples. The blue numbers in the upper side of the figure represent the mean daily weight gain (g) of the samples that are included in each of the four main branches of the clustering tree. The names in the x-axis at the bottom of the figure correspond to the code given to each lamb.

The effect of additive intake over the most abundant ASVs overall was evaluated and represented as a distribution heatmap in Figure 5, where samples were arranged depending on their similarity in the composition of these ASVs. A clear separation between the microbiota from control (green) and additive-diet lamb feces (red) can be observed. The blue numbers over the four main branches of the clustering tree indicate the value of the mean daily weight gain of the samples included in that branch of the tree. It can be observed the higher values in the mean daily weight gain in those parts of the heatmap that are mainly represented by additive-diet lamb fecal samples. ASV-1, ASV-3 and ASV-6, the most abundant ASVs of *Bifidobacterium*, *Veillonella* and *Lactobacillus*, respectively, were mainly represented in additive-diet lamb fecal samples. LEfSe revealed some *Bifidobacterium*, *Enterococcus* and *Lactobacillus* ASVs positively correlated with the additive-diet and drove the main contribution to the dissimilarity in bacterial community structures between the lambs fed with additive- or control-diets (Supplementary File 3). It can also be observed that some additive- and control-diet samples appear in the part of the graph that is mainly dominated by samples from the opposite group. Strikingly, two out of the three control-diet lambs that appear clustered with the additive-diet lambs showed a mean daily weight gain over the mean of control-diet lambs. Similarly, the additive-diet lambs that appear clustered with control-diet lambs had a mean daily weight gain below the mean for additive-diet lambs.

**Figure 6:**
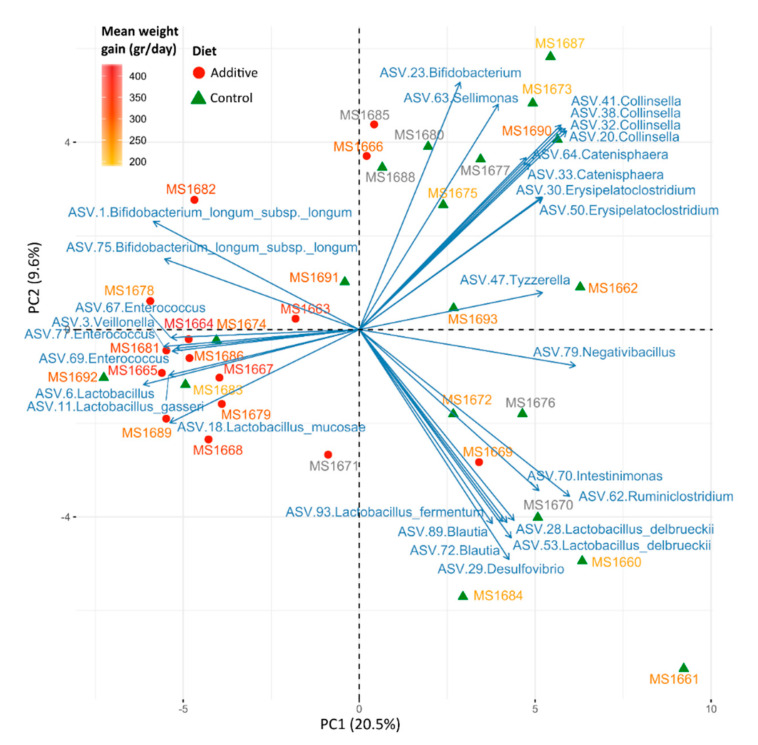
Principal Component Analysis (PCA) of the 100 most abundant ASVs in lamb feces. Sample dots are colored and shaped according to each lamb’s diet (red circles or green triangles for additive or control diets, respectively) and sample names are colored regarding the mean daily weight gain of that lamb (from yellow, lower, to red, higher). Grey names represent those samples in which the mean daily weight data is missing. The 30 ASVs that contributed mainly to the separation of the samples in the analysis are represented as blue arrows heading those samples where they were especially abundant.

The correlation between the most abundant ASVs, the additive intake and the mean daily weight gain was evaluated by Principal Component Analysis (PCA) and represented in Figure 6. A clear separation can be seen between high and low weight gain lamb samples, which correspond mainly to additive and control diets, respectively. The samples that clustered closer to their opposite group in Figure 5 can clearly be seen here. The ASVs that contributed mostly to the PCA are represented as blue arrows (heading those samples where they were more abundant) and showed some ASVs of *Bifidobacterium*, *Enterococcus*, *Lactobacillus* and *Veillonella* associated with higher mean daily weight gain lambs.

## 4. Discussion

The investigation of the ruminant gastrointestinal tract (GIT) microbiota is a main aspect, as it is responsible for feed digestion and exerts important physiological and immune functions [12]. GIT microbiota can be modulated through diet with specific aims, such as increased feed efficiency and host’s health and decreased gas emissions. This modification is particularly relevant in young ruminants, whose GIT microbiota is more susceptible to changes [1,8,13,22,23,54]. In this study, we supplemented lamb milk powder with a combination of two well-known prebiotics extracted from residues of the food industry—FOS from sugar beet and garlic residues (altogether named “additive”). The results showed that the consumption of this additive resulted in a significant increase in the mean daily weight gain of lambs.

The bacterial composition of lamb feces was evaluated by using 16S rRNA gene amplicon high-throughput sequencing (HTS). The results revealed that most samples were dominated by bacteria that are commonly found in ruminant GIT samples, such as *Bacteroides*, *Bifidobacterium*, *Lactobacillus*, *Escherichia* and *Veillonella* [17,18,21,55,56]. Strikingly, ASVs belonging to *Bifidobacterium*, *Lactobacillus* and *Veillonella*, that have been previously reported to benefit host’s health, were significantly associated with the additive intake. *Bifidobacterium* has been reported to be important for the early development of the GIT by actively influencing the regulation of the intestinal microbial homeostasis, the inhibition of pathogens, the modulation of local or systemic immune responses, the production of vitamins and the bioconversion of dietary components to bioactive compounds [13,57]. *Lactobacillus* has been previously linked to the increase of weight, improvement of the immune system and the increase of total serum IgG concentration in young calves [58]. However, despite the higher abundance of *Lactobacillus* in additive-diet lambs in our study, no significant differences were found between the two groups of lambs in any of the investigated serum immunoglobulins. Both *Bifidobacterium* and *Lactobacillus* are well-known beneficial microbiota, and constitute the major probiotics sold on the market [59]. There is evidence that the administration of these bacteria in young ruminants resulted in an increased weight gain and feed conversion ratio [60,61,62]. Therefore, based on the higher abundance of *Bifidobacterium* and *Lactobacillus* in additive-diet lamb feces, we are firmly convinced that they were the main drivers of the increased weight gain observed in this group of lambs. However, other bacteria might have also been involved in the increased weight gain of the additive-diet lambs. That is the case of *Veillonella*, that we found to be strongly linked to the additive intake. Previous studies have reported a positive effect of *Veillonella* over the increased weight of steers [63]. Therefore, the implication of *Veillonella* on the increased weight gain of the additive-diet lambs from this study cannot be ruled out.

To our knowledge, this is the first study that evaluates the effects of a combination of FOS and garlic over the weight gain of young ruminants and the changes that occur in their GIT microbiota based on their intake. This is a pilot study aiming to investigate the combination of the mentioned prebiotics altogether, constituting the actual prebiotic to test that was formulated ad hoc based on preliminary studies and the state-of-the-art in prebiotics for animal nutrition. Several studies have been previously developed in order to evaluate the effect of FOS or garlic independently on members of the commensal gut microbiota, which influenced the development of the “additive” investigated here. Therefore, the aim of the study was to evaluate the combination of FOS and garlic as a “unique prebiotic”. Previous studies reported that FOS had a positive effect over the weight gain of ruminants [34,35]. Moreover, several studies have reported the ability of certain bifidobacteria, such as *Bifidobacterium adolescentis*, *B. breve*, *B. infantis* and *B. longum*, and LAB, such as *Lactobacillus acidophilus*, *L. bulgaricus*, *L. lactis*, *L. plantarum* and *L. casei*, to selectively degrade FOS [64,65]. These results are in agreement with the increased relative abundance of *Bifidobacterium* and *Lactobacillus* members that we found in additive-diet lambs feces.

On the other hand, there is no evidence of garlic as a potential factor that increases animal weight gain [39]. Garlic is well-known as an ancestral medical plant due to its bactericidal capabilities against certain bacteria, as stated in the Introduction, and several studies have revealed that the ingestion of garlic powder influences the GIT microbiota. Filocamo et al. [38] analyzed the effect of garlic powder over different commensal members of the human gut commensal microbiota. They pointed out an increased resistance of *Lactobacillus casei* to the garlic powder in comparison to other bacteria, such as *Clostridium nexile,* and hence, their relative abundance increased. An older study from Cummings and Macfarlane [66] revealed that garlic fructans selectively enhanced bifidobacterial growth. Taking these two studies together, we can conclude that garlic might work as a double-edged sword for gut microbiota, by exerting a negative effect on potential pathogens without adversely affecting beneficial and commensal microbiota.

Although our results showed that the additive intake increased the weight of lambs that consumed it, several lambs from the control group also showed high weight gain values. Strikingly, the microbial composition in feces of this small section of the control group revealed high similarity with the bacteria identified in additive-diet lambs. This strengthen our hypothesis that the additive favors the growth of certain bacteria naturally present in the GIT of preweaned lambs, that could have similarly flourished in natural conditions, even though to a lesser extent.

The combinative administration of FOS and garlic might have an impact on ruminant health, as they have shown to limit the ability of potential pathogen growth and the incidence of diarrheal diseases. This could be particularly relevant in lamb farms where lambs are not fed with breast milk, as the ones from this study, considering the recent results obtained by Bi et al. [21] were they correlated bottle-feeding with an increased abundance of potential pathogens such as *Escherichia*/*Shigella* and diarrheal incidences. *Escherichia* is a well-known member of the normal intestinal microbiota of animals, and it comprises a very versatile genus including harmless commensal and pathogenic strains that can provoke intestinal diseases, such as diarrhea, which is the main cause of morbidity and mortality in newborn ruminants [21,61]. Some studies have reported the effect of FOS, *Bifidobacterium* and *Lactobacillus* in hindering the attachment of *Escherichia*, to the epithelial cell surfaces and thus reducing the incidence of diarrheal processes [29,67,68]. We identified a lower relative abundance of *Escherichia* in additive-diet lambs overall. However, during the development of this study, no significant diarrheal incidents occurred and therefore the effect of the additive over these situations might require further investigation.

For the microbiota composition analysis performed in this study, we used the deep taxonomic resolution provided by ASV resolution. ASVs offer deeper taxonomic resolution than classic Operational Taxonomic Units (OTUs) and constitute independent units that can be analyzed and compared across different datasets [69]. By using this approach, we were able to identify certain ASVs that were strongly linked to the additive intake and the increased weight gain. That is the case of *Enterococcus*, a LAB normally found in ruminants’ GIT and some of the species, such as *E. faecium*, has been shown to limit rumen acidosis [70,71] and to enhance forage digestibility and milk yields [72]. Even though no significant differences in the abundance of *Enterococcus* between the two groups of lambs were observed at the genera level, several ASVs were identified to be strongly linked to the additive intake and the increased weight gain. The possibility to identify bacteria at the subgenus level resolution provided by ASV identification can have important repercussions, such as that species or strains from the same genera may harbor different genetic pools [73,74].

Despite the use of advanced tools that face the state-of-the-art in microbial ecology, we are aware of the limitations of the methodology employed. We used 16S rRNA gene amplicon HTS, whose limitations have been stated elsewhere [75,76,77]. Further investigation accomplishing shotgun/metagenomic sequencing might be needed in order to deeply characterize the microbial communities and their genomic content. Additionally, we encountered some difficulties in assigning taxonomic profiles for certain ASVs, probably due to low representation of ruminant GIT microbiota in commonly used databases [78]. However, the new insights provided by this study coupled to the development of ongoing rumen-specific databases, such as the Hungate 1000 [79,80] and the metagenomic associated genomes (MAGs) extracted from rumen shotgun sequencing [81,82], may help to deepen our knowledge on ruminant-associated bacteria and to improve their taxonomic classification. Additionally, the microbial composition evaluation was performed over fecal samples due to their noninvasive nature, as lambs were sacrificed after the study for human consumption. Therefore, we could not investigate the long-term effects of the additive. Further development of this project might encompass a greater sample size, the evaluation of long-term effects and the evaluation of potential sources of microbiota, that have shown to be crucial in ruminant microbiota [21,83].

## 5. Conclusions

The administration of sugar beet FOS and garlic residues increased preweaned lamb weight gain and induced changes in their GIT microbiota, by enhancing the growth of several beneficial bacteria, such as *Bifidobacterium*, *Lactobacillus* and *Veillonella*. This study points out the combination of these two prebiotics as a potential approach in order to improve lamb production yields. Further studies encompassing functional HTS approaches, such as metagenomics and metatranscriptomics, may help to improve our knowledge in the relationship between prebiotics and GIT microbiota and to identify key organisms as a potential source of probiotics.

## Supplementary Materials

The following are available online at https://www.mdpi.com/2218-273X/10/8/1179/s1, File S1: Mean daily weight gain of lambs at the end of the study (25 days) and serum immunoglobulins concentration in lambs, File S2: Sequences per sample, alpha diversity indices and Good’s estimated sample coverage for 16S rRNA gene amplicons analyzed in this study, File S3: LEfSe analysis of the 100 most abundant ASVs found in lamb feces.
