# Supplementary material for: Dietary Supplementation with Sugar Beet Fructooligosaccharides and Garlic Residues Promotes Growth of Beneficial Bacteria and Increases Weight Gain in Neonatal Lambs
Source: Biomolecules. 2020 Aug 13;10(8):1179. doi: 10.3390/biom10081179 (PMC7465112; doi:10.3390/biom10081179)
Supplement: Supplementary file 1 [file biomolecules-10-01179-s001.zip › Supplementary-Material-883565.pdf]

Supplementary File 1. Mean daily weight gain of lambs at the end of the study (25 days) and serum immunoglobulins concentration in lambs. Differences were considered significant when p value < 0.05. SD = standard deviation.

	Mean	SD	Mean	SD	*p* value
	**Control-diet**		**Additive-diet**		
Mean daily weight gain	239	38.5	301	62	0.001
**Day 2**					
IgG (mg/ml)	7.8	3.55	7.87	3.968	0.991
IgA (mg/ml)	6.83	6.334	7.51	5.861	0.775
IgM (mg/ml)	1.87	0.958	2.21	0.633	0.22
**Day 16**					
IgG (mg/ml)	5.02	1.738	4.9	1.415	0.991
IgA (mg/ml)	23.76	10.591	20.59	8.909	0.283
IgM (mg/ml)	2.96	1.545	3.21	0.921	0.641

Supplementary File 2. Sequences per sample, alpha diversity indices and Good's estimated sample coverage for 16S rRNA gene amplicons analyzed in this study.

Description	Sequences per sample	Chao1	Shannon	Simpson	Observed ASVs	Good's coverage
Additive-diet lamb	112,749	153.56	4.46	0.91	153.56	1.00
Control-diet lamb	92,638	202.14	5.38	0.95	202.14	1.00

Supplementary File 3 LEfSe analysis of the 100 most abundant ASVs found in lamb feces. Differences in relative abundances of ASVs between additive- and control-diet lamb groups were considered when p value < 0.05. Taxonomy assignation was performed according to SILVA database.

LDA = Linear Discriminant Analysis

Attached document: Supplementary_File_3.Lamb-ASV-LEfSe.xlsx
